# demuxSNP: supervised demultiplexing single-cell RNA sequencing using cell hashing and SNPs

**DOI:** 10.1093/gigascience/giae090

**Published:** 2024-11-28

**Authors:** Michael P Lynch, Yufei Wang, Shannan Ho Sui, Laurent Gatto, Aedin C Culhane

**Affiliations:** School of Medicine, Limerick Digital Cancer Research Centre, Health Research Institute (HRI), University of Limerick, Limerick V94 T9PX, Ireland; Department of Cancer Immunology and Virology, Dana-Farber Cancer Institute, Boston, MA 02215, USA; Harvard Medical School, Boston, MA 02115, USA; Harvard T.H. Chan School of Public Health, Boston, MA 02215, USA; Computational Biology and Bioinformatics Unit (CBIO), de Duve Institute, Université catholique de Louvain, Brussels 1200, Belgium; School of Medicine, Limerick Digital Cancer Research Centre, Health Research Institute (HRI), University of Limerick, Limerick V94 T9PX, Ireland

**Keywords:** single-cell, demultiplexing, cell hashing, SNPs

## Abstract

**Background:**

Multiplexing single-cell RNA sequencing experiments reduces sequencing cost and facilitates larger-scale studies. However, factors such as cell hashing quality and class size imbalance impact demultiplexing algorithm performance, reducing cost-effectiveness.

**Findings:**

We propose a supervised algorithm, demuxSNP, which leverages both cell hashing and genetic variation between individuals (single-nucletotide polymorphisms [SNPs]). demuxSNP addresses fundamental limitations in demultiplexing methods that use only one data modality. Some cells may be confidently demultiplexed using probabilistic hashing methods. demuxSNP uses these data to infer the genotype of singlet and doublet clusters and predict on cells assigned as negative, uncertain, or doublet using a nearest-neighbor approach adapted for missing data.

We benchmarked demuxSNP against hashing, genotype-free SNP and hybrid methods on simulated and real data from renal cell cancer. demuxSNP outperformed standalone hashing methods on low-quality hashing data benchmark, improved overall classification accuracy, and allowed more high RNA quality cells to be recovered. Through varying simulated doublet rates, we showed that genotype-free SNP and hybrid methods that leverage them were impacted by class size imbalance and doublet rate. demuxSNP’s supervised approach was more robust to doublet rate in experiments with class size imbalance.

**Conclusions:**

demuxSNP uses hashing and SNP data to demultiplex datasets with low hashing quality where biological samples are genetically distinct. Unassigned or negative cells with high RNA quality are recovered, making more cells available for analysis. Data simulation and benchmarking pipelines as well as processed benchmarking data for 5–50% doublets are publicly available. demuxSNP is available as an R/Bioconductor package (https://doi.org/doi:10.18129/B9.bioc.demuxSNP).

## Introduction

Single-cell RNA sequencing (scRNA-seq) enables insight into cellular heterogeneity, cell subtypes, and cell–cell communication not previously possible with bulk methods due to gene expression averaging [1]. Cost remains a barrier for large-scale research and clinical studies at a single-cell resolution [2] despite reductions in the cost of sequencing technologies. Multiplexing in scRNA-seq refers to the sequencing of cells from multiple different biological samples on the same sequencing lane rather than on individual lanes. This reduces sequencing costs and technical batch effects [3]. The cells must then be demultiplexed, or assigned back to their biological sample of origin prior to downstream analysis. In droplet-based technologies, higher cell loading rate results in a higher doublet rate (two or more cells captured in a single droplet), thus limiting the lane capacity. In multiplexed experiments, doublets made up of cells from different samples are more easily identified and removed, allowing higher cell loading rate onto the sequencing lane. Demultiplexing strategies broadly follow two approaches, experimental cell tagging (cell hashing) and bioinformatics analysis of genetic variation using single-nucleotide polymorphisms (SNPs). Cell hashing is popular due to its applicability to a wide variety of experimental designs and availability of commercial hashing kits. SNP-based methods are limited to genetically distinct samples but have lower library preparation costs.

Cell hashing is a combined experimental and computational approach where cells from each biological sample are labeled with a distinct sequenceable tag [4, 5] prior to being pooled and sequenced. Computational algorithms, such as those reviewed by Howitt et al. [6], then operate on the resulting counts matrix to determine which cells came from which biological sample of origin. However, technical artifacts such as nonspecific binding, doublets, and varying cell quality due to cell stress may complicate this procedure. Cells with low hashing quality may be assigned to the incorrect group. Additionally, cells deemed to have no hashing signal in any group remain unassigned and are referred to as hashing negatives, or negatives for short. Small numbers of hashing negatives are permissible, but large numbers of negatives result in wasted data and so are undesirable. Negative cells that cannot be assigned are removed prior to downstream analysis steps, resulting in wasted data. Additionally, researchers may also exclude cells if there is disagreement between demultiplexing algorithms or low assignment probability. This results in further wasted data and reduces the effectiveness of multiplexing as a cost-saving measure. Alternatively, retaining uncertain cells that may be wrongly assigned reduces the statistical power of differential gene expression analysis and confounds biological interpretations in downstream analysis steps. Due to their dependence on hashing quality, performance of standalone hashing-based demultiplexing methods can vary significantly between datasets [7].

SNP-based demultiplexing methods exploit natural genetic variation between genetically distinct biological samples. Genotype-based methods such as Demuxlet [8] and scSNPdemux [9] require *a priori* knowledge of the genotype of each biological sample, incurring additional experimental cost and limiting their utility. Genotype-free methods [10–12] do not require *a priori* knowledge of sample genotypes, so they are more commonly used but also face limitations. Although they can group cells, they cannot link cells back to a biological sample without additional genotype or hashing data. Calling SNPs in scRNA-seq is challenging as the data are sparse, with reads concentrated in specific regions, and gene expression can be highly variable within a dataset [13]. Performance reduces in datasets with high levels of ambient RNA [14]. Despite the considerable number of methods available, a universally robust tool has yet to be developed.

Some recent methods use both SNP-based and hashing modalities. HTOreader [15, 16] performs hashing demultiplexing using a mixture model approach, which is then integrated with hashing results from existing genotype methods, allowing an increased overall cell recovery rate and recovery of up to one missed hashing group. hadge [17] runs a selection of existing genotype and hashing algorithms and finds the pair of methods across modalities with the highest correlation. We developed a supervised multimodal method, demuxSNP, that leverages both genotype and hashing modalities along with a Nextflow pipeline [18] to benchmark against existing standalone hashing (HTODemux [4, 19], BFF_raw and BFF_cluster [20, 21], GMM-Demux [22, 23], demuxmix [24, 25]) and genotype-free SNP-based (souporcell [12, 26]) and hybrid (HTOreader [15, 16]) methods, adapting published SNP simulation pipelines [14] paired with hashing data, to better understand performance across a range of scenarios against reliable ground truth [18]. We further motivate the utility of demuxSNP over popular existing methods with application to a case study renal cell cancer dataset. demuxSNP is available as an R/Bioconductor package [27].

## Results

### Overview of demuxSNP

**Figure 1:**
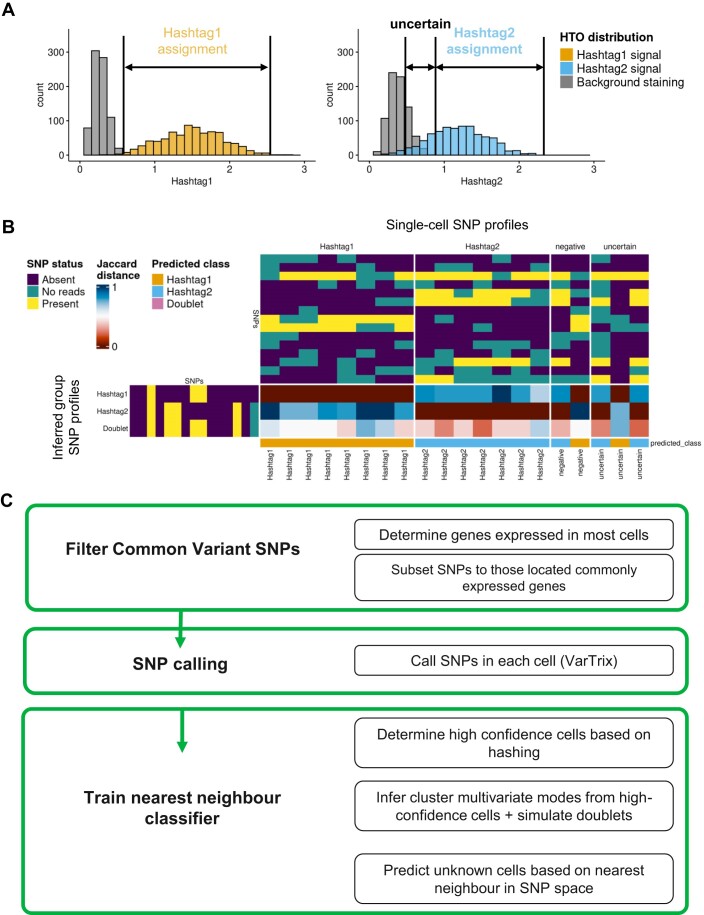
Overview of the demuxSNP workflow. (A) High-quality hashtag counts can be separated into a bimodal distribution with distinct signal and background peaks. Low-quality hashtag counts have a poorly separated bimodal distribution and have high numbers of misassigned, uncertain, or hashing negative cells. (B) SNPs called in single cells contain missing data and noise. To improve signal, singlet and doublet cluster SNP profiles can be inferred. Cells assigned as uncertain, negative, or doublet can be compared against inferred SNP profiles and classified using a nearest-neighbor approach. (C) demuxSNP workflow.

A key challenge in hashing-based demultiplexing is variability in hashing quality due to technical issues such as nonspecific binding. In general, a proportion of cells from each group may be confidently called, while some may remain uncertain or negative for a signal, the number of which will depend on the hashing quality of a specific experiment (Fig. 1A). These high-confidence cells may be identified using consensus methods such as cellhashR [20, 21], probabilistic methods with a high acceptance threshold [22, 24], or use of a nonconservative count threshold to describe the positive peak; however, retaining only these high-confidence cells results in loss of valuable data through negative or uncertain cells.

For the cells that cannot be confidently called using hashing methods, we propose that their correct group may be more easily identified based on their SNP profile. We apply demuxmix [24], a highly performant probabilistic demultiplexing algorithm to hashing counts data to determine which cells can be confidently called. SNPs are called in single cells, and the SNP profile of singlet and doublet groups may be inferred from the high-confidence singlets. The class of uncertain, negative, or doublet cells is then determined based on their most similar SNP profile using Jaccard distance (Fig. 1B) adapted for missing data. With high-quality hashing data, often a large proportion of cells can be called with high confidence. With low-quality hashing data, significant numbers of cells may be assigned as negative or uncertain and their recovery warranted using a method such as demuxSNP. Summary statistics from 12 datasets demultiplexed with HTODemux show percent negatives range from 1–17% (Supplementary Table S1), although values significantly higher have been reported in other benchmarking studies [7, 6]. The demuxSNP workflow is outlined in Fig. 1C.

### Demultiplexing performance improves when using demuxSNP compared to standalone hashing methods on datasets with low hashing quality

**Figure 2:**
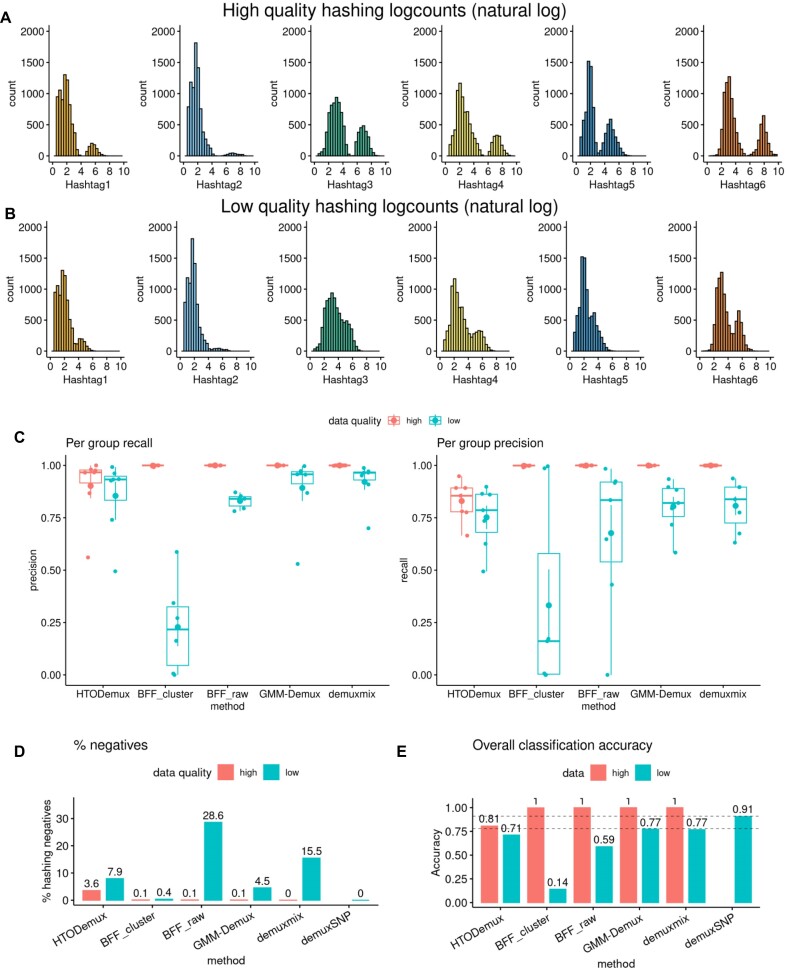
demuxSNP improved cell assignment on datasets with low hashing quality. (A) Hashing logcounts (natural log) for benchmarking high-quality hashing. The signal and background are distinct. (B) Hashing logcounts (natural log) for benchmarking low-quality hashing. There is poor separation between signal and background. (C) Hashing algorithm precision and recall decreased with hashing quality. (D) Low-quality hashing resulted in large numbers of hashing negative cells. (E) demuxSNP increased overall classification accuracy on low-quality hashing data compared with standalone hashing methods.

Simulated data allow for comparison against a reliable ground truth for different experimental and technical configurations. Benchmark data are simulated from a multiplexed experiment with six hashtags from genetically distinct samples. Aligned reads and hashing counts from singlets assigned with high confidence by demuxmix [ 24, 25] are retained. Doublets are simulated from the singlet data by randomly renaming barcodes on aligned reads [14] and summing counts across singlet cells comprising each doublet for SNP calling and hashing data, respectively [18]. Hashing quality is reduced by scaling down the signal in each hashtag group. Features associated with high-quality hashing include well-separated bimodal peaks and a high signal-to-noise ratio (Fig. 2A). Other experimental factors that may improve demultiplexing performance include well-balanced group sizes. Low hashing quality is then associated with features such as poor peak separation and low signal-to-noise ratio, with high class imbalance also impacting demultiplexing performance (Fig. 2B).

We compared demultiplexing performance of several popular hashing-based algorithms and observed that performance decreased on low-quality hashing compared to high-quality hashing regardless of method, shown here on hashing data with a typical doublet rate of 20% (Supplementary Table S1). On the high-quality dataset, HTODemuxscored lower than other methods tested for both precision and recall, which may be attributed to features other than peak separation such as imbalance in class sizes and the misalignment of the signal peaks (Fig. 2C). Other methods, BFF_raw, BFF_cluster, GMM-Demux, and demuxmix, each showed high precision and recall on the high-quality dataset, an expected result given the clear separation between signal and background. On the low-quality dataset, BFF_raw performed poorly, potentially due to the assumption of a bimodal distribution, the extent of which is reduced in this benchmark.

We next explored which methods recovered more cells and thus had fewer cells with no identity (hashing negatives). In terms of the number of assigned hashing negatives (Fig. 2D), on the high-quality dataset, HTODemux assigned the most negatives (∼3.6%). Few (<0.2%) negatives were assigned by BFF_raw, BFF_cluster, GMM-Demux, and demuxmix. On the low-quality dataset, BFF_raw and demuxmix assigned the most negatives (28% and 15%, respectively). demuxSNP avoids the classification of hashing negatives by leveraging SNP data to assign these cells, reducing wasted data. BFF_cluster assigned the fewest negative cells, but we note that while classifying few hashing negatives is a desirable attribute, this reflects only one aspect of algorithm performance and must be taken in the context of other classification performance metrics.

We finally looked at overall classification accuracy (Fig. 2E). Each of the standalone hashing algorithms, with the exception of HTODemux, performed almost perfectly on the high-quality dataset. We did not compare demuxSNP on this dataset as a large number of cells (∼99%) had already been confidently called by standalone probabilistic hashing algorithms, and thus the use of demuxSNP was not warranted. On the low-quality dataset, mixture models GMM-Demux and demuxmix outperformed other standalone hashing methods. Despite assigning fewer negatives, BFF_cluster had the lowest overall accuracy, again potentially due to the assumption of a bimodal distribution. Performance improved when using hashing and SNPs to assign cells compared to hashing classification alone, with overall classification accuracy of 0.91 for demuxSNP compared to 0.77 and 0.77 for the top-performing standalone hashing methods, GMM-Demux and demuxmix, respectively.

### demuxSNP is more robust to class size imbalance compared to genotype-free SNP method souporcell and hybrid method HTOreader

**Figure 3:**
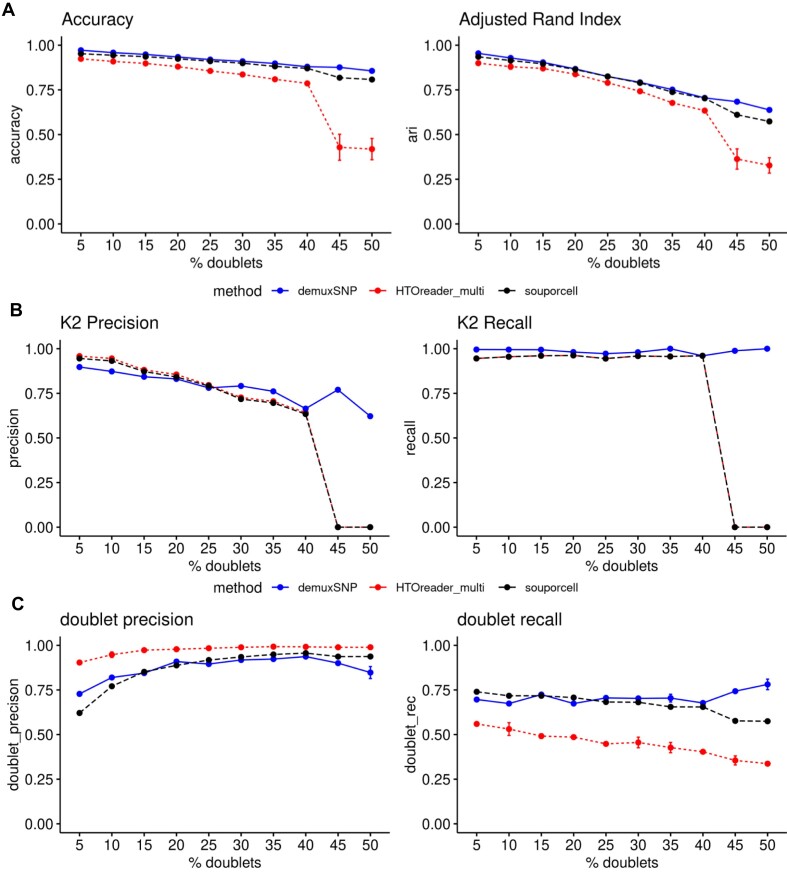
Comparison of hybrid and SNP-based methods. (A) demuxSNP (average of 5 runs ± SD) outperforms souporcell (single run, seed fixed) and HTOreader (average of 5 runs ± SD) for overall classification accuracy. (B) Precision and recall for classifying the minority cluster (K2). demuxSNP performance remains stable. (C) Precision and recall for classifying doublets (multisample).

We next benchmarked demuxSNP against the standalone genotype-free SNP-based method souporcell and hybrid method HTOreader. souporcell classifies cells using a sparse mixture model. HTOreader first fits a Gaussian mixture model to the hashing counts and then integrates the hashing results with results from third-party SNP-based methods—in this case, souporcell. We first benchmarked overall classification performance in terms of accuracy and adjusted Rand index (ARI) across a range of doublet rates from 5–50% [28] (Fig. 3A). Here, demuxSNP slightly outperformed souporcell, with greater differences observed at doublet rates over 40%. HTOreader slightly underperformed at low doublet rates (5–40% doublets), but performance dropped considerably at high doublet rates (45–50% doublets).

In evaluating the performance of clustering methods on scRNA-seq gene expression data, significant attention is given to methods’ ability to detect small clusters [29]. Genotype-free SNP-based methods face similar challenges in clustering genetically distinct SNP profiles, where the number of cells per biological sample may vary and doublets may obscure the signal. We observed in a case study dataset that at high doublet rates, the minority cluster (K2) appeared to be completely misassigned by souporcell, and we replicated this in our benchmarking (Supplementary Fig. S1A). The true K2 cells were assigned as doublets, while the cells assigned as K2 were true doublets. In contrast, demuxSNP correctly identified the K2 group (Supplementary Fig. S1B). The assignment of a large proportion of doublets to a singlet group has the potential to confound downstream analysis, if not identified.

To investigate this further, we systematically tested whether doublet rate impacted imbalanced classification and whether demuxSNP’s supervised approach was more robust to assigning minority clusters when doublet rate increased compared to unsupervised methods. At lower doublet rates, demuxSNP, souporcell, and HTOreader performed comparably. However, at higher doublets rates (over 40%), both the precision and recall for souporcell and HTOreader reduced to zero (Fig. 3B). demuxSNP’s performance remained stable.

Hybrid methods such as HTOreader and hadge can leverage genotype-free SNP-based methods, and so we next asked whether errors in genotype-free methods’ assignments would impact hybrid methods. HTOreader, using souporcell’s results for hybrid classification, reduced in performance at the same threshold as souporcell (Fig. 3A, B). When applied to the renal cell cancer dataset, we observed that this misassignment of a singlet cluster by souporcell resulted in a propagation of mismatches between hashing and SNP clusters when HTOreader integrated these results (Supplementary Fig. S2) as a one-to-one match does not exist, explaining the significant drop in overall performance observed in Fig. 3A compared to souporcell.

### SNP-based and hybrid methods assign multisample doublets with high precision and low recall

The ability to correctly identify doublets remains a challenge and has important implications for downstream analysis and experimental design considerations such as cell loading rate. In the context of demultiplexing, we differentiate between multisample doublets (containing cells from two or more different samples and generally referred to simply as doublets in the context of demultiplexing) and single-sample doublets (containing cells from only a single sample). Both may confound biological interpretation of the data if not removed, but only multisample doublets may be identified and removed by demultiplexing methods. The percentage of multisample to single-sample multiplets is related to the number of samples multiplexed (Supplementary Fig. S3A). In experiments with few multiplexed samples, a high percentage of the total doublets will be single-sample and not identifiable with demultiplexing. Conversely, for highly multiplexed experiments, most doublets will be multisample and so could, in theory, be removed using demultiplexing and further doublet removal steps not required or become less critical.

To test the doublet detection capabilities of different methods, we first calculated the numbers of doublets assigned against the true number of multisample doublets across each dataset. Typically, methods underclassified (multisample) doublets at a rate roughly proportional to the overall doublet rate (Supplementary Fig. S3B). We further tested the doublet precision and recall for different SNP-based and hybrid methods. We observed higher precision and lower recall across methods, with HTOreader generally scoring highest precision but lowest recall. Overall, SNPs and hybrid methods rarely classified singlets as doublets but often labeled doublets (multisample) as singlets. Although the doublet recall remains approximately constant, the negative impact of this increases with doublet rate and explains the reduced accuracy and ARI in Fig. 3A as doublet rate increases.

### demuxSNP overcomes demultiplexing challenges in case study dataset

**Figure 4:**
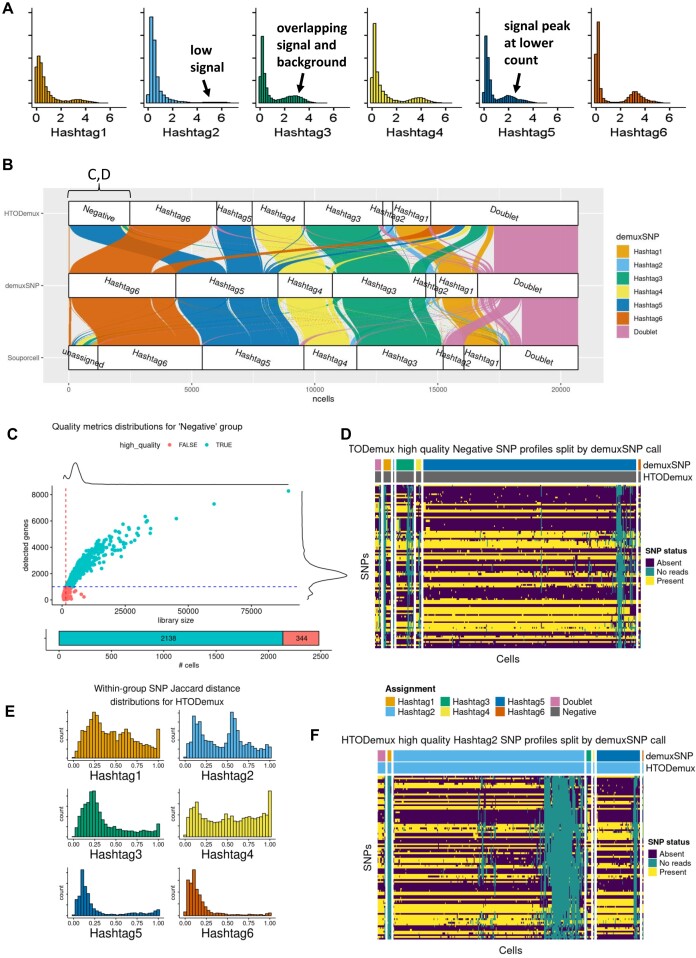
demuxSNP overcomes demultiplexing challenges on real data. (A) Hashing data contained features of low-quality hashing such as low signal, low signal-to-noise ratio, and unaligned signal peaks. (B) Most negatives called by HTODemux were assigned as Hashtag5 by demuxSNP and souporcell. Majority of Hashtag2 called by souporcell was called doublet by demuxSNP and HTODemux. (C) Quality metric distribution for HTODemux negative group. (D) SNP profiles of HTODemux negative group. (E) Distribution of binary distance matrix for HTODemux singlet group SNP profiles. (F) SNP profiles of HTODemux Hashtag2 group showed multiple SNP profiles.

We next demonstrate the utility of demuxSNP on a case study dataset containing cells from six genetically distinct samples from renal cell cancer. We identified features in the hashing counts indicating low hashing quality (Fig. 4A), including low signal-to-noise ratio (Hashtag 3,5), low signal (Hashtag2), and misaligned peaks (Hashtag5). We applied HTODemux (a popular hashing-based method), souporcell (a popular genotype-free SNP-based method), and demuxSNP and observed significant disagreement between assignments. Notably, HTODemux assigned a large number of hashing negative cells, and souporcell showed little agreement with HTODemux and demuxSNP in the Hashtag2 group (Fig. 4B).

We observed poor agreement between HTODemux, demuxSNP, and souporcell in the Hashtag2 group. Most cells assigned as Hashtag2 by HTODemux and demuxSNP were assigned to Hashtag4 or the doublet group by souporcell. A large number of cells (*n* = 1,043) assigned to the Hashtag2 group were consistently called doublets by demuxSNP and HTODemux, leading to significant potential for confounding downstream analysis steps. This is consistent with the behavior explored in Fig. 3B, where souporcell was unable to identify the minority cluster in datasets with high doublet rates. demuxSNP successfully identified the minority cluster due to its supervised classification approach.

A large number of cells (*n* = 2,582, >10% of the dataset) were assigned to the negative group by HTODemux, meaning that they could not be assigned due to their hashing quality. It was previously identified that cells with low hashing counts (negative) also had low RNA quality [4], and so we next asked whether these negative cells were truly low-quality cells. We plotted standard quality control metrics, library size, and number of detected features for each negative cell. We observed that the majority (2,138 out of 2,482, 86%) pass standard scRNA-seq quality checks (Fig. 4C). We visualized SNP profiles from the HTODemux negative group, coloring cells by the HTODemux and demuxSNP classification and splitting by the demuxSNP classification, and observed a consistent SNP profile in each reassigned group. Most cells were reassigned to Hashtag5, consistent with the souporcell and demuxSNP annotations. We compared the dissimilarity of the reassigned cells to the SNP profile of the sample they were assigned to using Jaccard distance (Supplementary Fig. S4A). A clear signal can be observed with the lowest distance between reassigned cells and the corresponding SNP profile.

We examined binary distance distributions of singlet group SNP profiles. The presence of a multimodal or bimodal distribution indicated cells from multiple biological samples (Fig. 4E). Singlet groups where a bimodal distribution was evident tended to have fewer cells called the same by HTODemux and demuxSNP. We observed highest proportions of agreed assignments between HTODemux and demuxSNP on hashtags with unimodal SNP distance distributions at 0.86, 0.75, and 0.90 for Hashtags 1, 2, and 4 compared to 0.92, 0.96, and 0.94 for Hashtags 3, 5, and 6, respectively. We visualized the SNP profiles of HTODemux Hashtag2, the group with poorest agreement between HTODemux and demuxSNP, and observed multiple SNP profiles. The main SNP profile was called consistently Hashtag2 by both HTODemux and demuxSNP. The remaining cells were reassigned by demuxSNP, mostly to Hashtag5 (Fig. 4F). Again, we compared the similarity of SNP profiles of the reassigned cells with the inferred SNP profiles (Supplementary Fig. S4B). The majority of cells showed the most similarity to Hashtag2, followed by other hashtags to a lesser extent. By leveraging both SNP and hashing modalities, demuxSNP increased the number of assigned cells that would have otherwise been labeled as negatives, as well as reassigning cells misassigned due to hashing quality.

## Discussion

Multiplexing is primarily a cost reduction measure now used in most single-cell experiments, allowing greater utilization of high-throughput assays. However, large numbers of negative, uncertain, or misassigned cells resulting from suboptimal demultiplexing reduce its effectiveness. Accurate assignment of cells to their original sample through demultiplexing is critical to interpretation of downstream analysis, minimizing wasted data through misclassified or unclassified cells, as well as maintaining confidence in the technique as a cost-saving measure to allow larger-scale experiments. To this end, we make key contributions compared to existing hashing- and SNP-based methods.

The dependence of hashing demultiplexing performance on hashing quality has been reported previously [20,6], yet many current solutions to this problem have focused on more advanced modeling of the counts data to optimize detection of signal from noise or consensus-type approaches. We proposed a novel method applicable to genetically distinct samples utilizing cell hashing and SNPs, overcoming dependence on hashing data quality by assigning hashing negative, uncertain, or doublet cells based on their SNP profiles. This results in overall improvements in classification performance, as well as assignment of hashing negatives, which may consist of a considerable proportion of the data.

We showed systematic biases in genotype-free SNP-based methods such as souporcell and the implications for hybrid methods that utilize them. Despite being performant in many scenarios [30], souporcell’s unsupervised classification results in systematic flaws in identifying minority clusters at high doublet rates, misclassifying doublets in place of the minority singlet group. While suggested as a potential limitation of a similar method previously [10], we believe this is the first time the problem has been described in greater detail. Additionally, errors in misassigning minority clusters such as this impact hybrid methods such as HTOreader, which leverage third-party SNP-based methods for classification. HTOreader was shown to be beneficial for demultiplexing in the case of a missing hashtag group [15], an application where it may be preferred over demuxSNP, as demuxSNP requires hashing to infer the cluster SNP profiles. However, we showed that demuxSNP is generally more performant, specifically in situations where genotype methods misassign a sample. Our benchmarking results suggest a threshold of 40% doublets after which minority clusters are missed, but this is likely dependent on other experimental factors and warrants future benchmarking. For example, we observed misassignment of a minority cluster by souporcell in the application dataset with an estimated 16–24% doublets based on the number of recovered cells [31]. We went on to show that the demuxSNP’s supervised method is more robust to doublet rate and class size imbalance.

Challenges persist for SNP-based and hybrid demultiplexing in terms of doublet identification, with a higher doublet rate resulting in reduced overall performance for demuxSNP, souporcell, and HTOreader. Both SNP-based and hybrid methods consistently show high precision and low recall for classifying doublets, indicating that downstream doublet detection remains a necessary step even if the ratio of multisample to single-sample doublets is high, particularly when incorporating an experimental design targeting a high cell loading rate.

demuxSNP provides a framework for how both SNP and hashing data can be combined to optimize demultiplexing. As a result, an obvious limitation then exists that the method is only applicable to genetically distinct biological samples, and so cross-validating demultiplexing results from genetically similar biological samples remains a challenge in the field. Unlike other SNP-based methods [10, 12], due to the use of binary distance measures, demuxSNP’s algorithm does not distinguish between homozygous and heterozygous SNP loci, and so it is not suitable for applications where heterozygosity is important such as discriminating between closely related genotypes. Additionally, as demuxSNP relies on hashing data to infer the SNP profile of each sample, this places an upper limit on the number of multiplexed samples where it can be used to demultiplex. As single-modality SNP-based methods do not require hashing, they allow for greater sample multiplexing to be considered during experimental design.

More generally, benchmarking hashing demultiplexing methods in scRNA-seq poses many challenges. First, defining ground truth is nontrivial. In the absence of a tool to simulate realistic hashing data, SNP-based demultiplexing methods have been used to define ground truth to benchmark hashing methods [6, 7]. Consequently, any biases inherent in the SNP-based method will be reflected in the benchmarking results. Second, and following on from this, it is not feasible to generate sufficient benchmarking datasets to evaluate changes in experimental conditions such as doublet rate, number of samples, and sample imbalance. This results in conclusions that are difficult to generalize, seen particularly in differing evaluations of the performance of Seurat HTODemux function between benchmarking studies [6,7]. In contrast, for benchmarking of SNP-based methods, strategies to simulate SNPs from real data have been developed elsewhere [14] and have been successful in evaluating the impact of factors such as doublet rate and ambient RNA content. This furthers the need for tools that allow realistic simulation of hashing counts, such as those used for simulating scRNA-seq data [32] to make more comprehensive benchmarking studies feasible.

The full impact of demultiplexing errors in published studies is difficult to estimate. Repositories for high-throughput sequencing data, including dbGaP, often require raw data such as FASTQ files to be submitted on a per-sample basis. For single-cell datasets that are typically multiplexed, this means that most data published in repositories are post-demultiplexing, and it is not possible to reproduce and reanalyze the scRNA-seq demultiplexing steps. Given the widespread use of multiplexing in scRNA-seq, this means most published studies are not fully reproducible. Additionally, in light of the limitations of existing demultiplexing methods we have reported, it is possible published data may include significant errors in cell assignment. The opportunity to quality control (QC) or reevaluate the integrity of the demultiplexing solution retrospectively is lost as the multiplexed data are not published. Furthermore, by only publishing demultiplexed data, which may have excluded large numbers of cells that were unassigned, there is considerable loss of valuable scRNA-seq data to the community.

A recent development that acknowledges the role multiplexing plays in the future of single-cell studies was the release of the “multi” functionality in CellRanger [33], the bioinformatics pipeline used to analyze data from the popular 10X Genomics single-cell platform using a Gaussian mixture model. While the exact model used has not, to our knowledge, been independently benchmarked, mixture model-type methods have shown to be more consistently performant in this study and elsewhere. However, this approach may come with some disadvantages. Previously, demultiplexing was carried out as part of downstream analysis, where the hashing quality could be reviewed and visualized and different algorithms tested. The incorporation of this step within the CellRanger pipeline will streamline downstream analysis but may consequently impede recovery of negative cells or identification of misassigned cells. Ongoing efforts to optimize laboratory protocols and workflows [34, 35] to improve data quality or alternative labeling technologies [36] less susceptible to nonspecific binding will be key in resolving this.

## Conclusion

Overall, we have shown that a multimodal framework allows demuxSNP to recover hashing negative cells, reassign cells miscalled by hashing algorithms based on their SNP profile, and overcome class size imbalance and doublet rate issues incurred by genotype-free SNP-based methods and hybrid methods that leverage them. The workflow has been implemented in the R/Bioconductor package demuxSNP, providing additional functionality for assisting in SNP selection and selecting training data. The package provides interoperability with the Bioconductor SingleCellExperiment class.

## Methods

### demuxSNP workflow

1. SNPs are filtered to those located within genes expressed across most cells in the dataset (optional).
2. VarTrix [37] uses the filtered SNP list to call SNPs in each cell.
3. Probabilistic hashing methods are leveraged to determine high-confidence singlets.
4. Labels from high-confidence singlets are used to infer multivariate mode per group and train a nearest-neighbor classifier based on adapted Jaccard distance and predict negative, uncertain, and doublet cells.

### Adapting Jaccard binary distance metric for missing data

Jaccard distance is a common distance measure that can be applied to binary data. Where *n* is the contingency matrix between two binary vectors and *a = n*_11_, *b = n*_01_, and *c = n*_10_, then the Jaccard index *j* is


\begin{eqnarray*}
j = a/\left( {a + b + c} \right).
\end{eqnarray*}

This can be computed using the matrix product where *m* is the binarized matrix for SNP locations supporting the alternative allele such that


\begin{eqnarray*}
a &=& m\ x\ {m}^T,\\
b &=& \left( {1 - m} \right)x\ {m}^T,\\
c &=& m\ x\ ( {1 - {m}^T} ).
\end{eqnarray*}

The standard implementation of Jaccard distance does not take into account missing values—in this case, whether a read was present at a given SNP location. To account for this, we perform an additional element-wise multiplication step on each side of the matrix product such that locations where no SNP read is present in either of the two vectors being compared are not counted. The above can be adapted to remove missing data where $p$ is the binary matrix for whether there are reads at a given SNP location such that


\begin{eqnarray*}
a &=& ( {m*p} )\ x\ ( m*p)^T,\\
b &=& ( {( {1 - m} )*p} )\ x\ ( {m*p)^T} ,\\
c &=& ( {m*p} )\ x\ ( {( {1 - m} )*p)^T} .
\end{eqnarray*}

To further mitigate the impact of missing data on classification, the multivariate mode is computed for each sample to infer a more complete SNP profile. Doublet profiles are calculated from singlet SNP profiles. As we calculate the distance between each cell to be predicted and the inferred centroids (training data), rather than between all cells, the final implementation looks like


\begin{eqnarray*}
a &=& \left( {{m}_{\textit{train}}*{p}_{\textit{train}}} \right)\ x\ {\left( {{m}_{\textit{predict}}*{p}_{\textit{predict}}} \right)}^T,\\
b &=& \left( {\left( {1 - {m}_{\textit{train}}} \right)*{p}_{\textit{train}}} \right)\ x\ {\left( {{m}_{\textit{predict}}*{p}_{\textit{predict}}} \right)}^T,\\
c &=& ( {{m}_{\textit{train}}*{p}_{\textit{train}}} )\ x\ {( {( {1 - {m}_{\textit{predict}}} )*{p}_{\textit{predict}}} )}^T.
\end{eqnarray*}

SNPs may be filtered to reduce computational cost. We provide additional data to show robustness to subsetting to SNPs within most commonly expressed genes (Supplementary Fig. S5). VarTrix [37] was used in consensus mode to call SNPs in single cells with default settings. High-confidence cells were determined using demuxmix with an acceptance threshold of 0.75. Classes of cells denoted as uncertain, negative, or doublet were then predicted using nearest neighbors.

## Datasets

### Single-cell RNA sequencing of renal cell cancer dataset

Single-cell RNA-seq experiments were performed by the Brigham and Women’s Hospital Center for Cellular Profiling. Sorted cells were stained with a distinct barcoded antibody (Cell-Hashing antibody, TotalSeq-C; Biolegend). After washing, the stained cells were resuspended in 0.4% bovine serum albumin in phosphate-buffered saline at a concentration of 2,000 cells per μL, then loaded onto a single lane (Chromium chip K; 10X Genomics), followed by encapsulation in a lipid droplet (Single Cell 5′ kit V2; 10X Genomics), followed by cDNA and library generation according to the manufacturer’s protocol. A 5′ mRNA library was sequenced targeting an average of 50,000 reads per cell, and a protein (hashtags) library was sequenced to an average of 15,000 reads per cell, all using Illumina Novaseq.

### Simulated datasets

#### Data preparation

Ground truth was obtained from a multiplexed renal cell cancer experiment by applying demuxmix [24] with a high acceptance threshold to generate a list of barcodes associated with each sample. From this, an individual bam file was generated per group using subset-bam [38], which formed the basis for SNP simulation.

#### SNP simulation

For the aligned reads, we followed the simulation strategy of Weber et al. [14] leveraging samtools [39]. Briefly, beginning with a single bam file per biological sample, a suffix was added to each cell barcode to identify cells from that group. The bam files were then merged. To simulate doublets, a lookup file was generated whereby randomly selected barcodes from a fixed number of cells were each renamed to the barcode from a different cell.

#### Hashing simulation

Low-quality hashing/uncertain cells were removed as part of the data preparation step. Using the same lookup file generated in the previous step, RNA and hashing counts for each doublet pair described in the lookup file were merged, and the sum of their respective RNA and hashing counts was retained. To replicate low-quality hashing data, the hashing signal was scaled down.

Percent simulated doublets included both single-sample and multisample doublets. For the purposes of measuring demultiplexing performance, single-sample multiplets were considered singlets and multisample multiplets considered doublets, as demultiplexing methods are only capable of identifying multisample doublets, and single-sample doublets are indistinguishable from true singlets. Data simulation steps and analysis are incorporated into an adaptable and reproducible Nextflow [40] pipeline.

### Demultiplexing summary statistics

Number of singlets, doublets, negatives, their percentages, and number of multiplexed samples were recorded from 14 recent datasets from the Harvard T.H. Chan School of Public Health Bioinformatics Core. Hashing demultiplexing was carried out using HTODemux. SNP demultiplexing was carried out using Freemuxlet.

### Benchmarking methods

souporcell [12, 26] was applied to the case study and simulated datasets using 1000 Genomes common variants [10], skipping remapping with default parameters. For direct comparison in simulated benchmarking, the filtered SNP list, as generated by souporcell, was used as input for demuxSNP. cellhashR [20, 21] “GenerateCellHashingcalls” was used to accommodate use of multiple algorithms [4, 20, 22, 24] and ensure consistency across preprocessing.

### Renal cell cancer case study

demuxSNP was applied using common variants from 1000 Genomes with >5% frequency filtered to SNPs located within the top 100 commonly expressed genes. souporcell was applied using default parameters and common variants supplied from 1000 Genomes common variants with >5% frequency. Hashing data were normalized using the centered log ratio method from NormalizeData() and demultiplexed using HTODemux with default parameters. Low-quality cells were determined as those with fewer than 1,500 UMIs (unique molecular identifiers) per cell and 1,000 genes per cell.

Visualization of SNP profiles within and between groups provided a useful assessment of whether misassigned cells are present in the data. Plotted as a heatmap, distinct SNP profiles appeared within groups. Quantifying the genetic variability within each assigned group allowed for assessment of the demultiplexing results. We used the “vegdist” function from the vegan [41] package to calculate the binary Jaccard distance between cells within the same assigned group. Homogeneous groups (containing mostly cells from a single sample) appeared unimodal, whereas groups containing misassigned cells from different groups were more heterogeneous and appeared multimodal.

Plots were generated using ComplexHeatmap [42], ggpubr [43], and ggalluvial [44].

## Availability of Source Code and Requirements

1. Bioconductor:

Project name: demuxSNP

Project homepage: https://doi.org/doi:10.18129/B9.bioc.demuxSNP [27]

Operating system(s): Windows, MacOS, Linux

Programming language: R

Other requirements: VarTrix

License: GNU GPL 3.0

biotoolsID: demuxSNP

RRID: SCR_025,703

DOI: https://doi.org/doi:10.18129/B9.bioc.demuxSNP

2. Workflow:

Project name: demux-doublet-simulation

Project homepage: https://workflowhub.eu/workflows/1160 [18]

Operating system(s): Linux

Programming language: Nextflow, Bash, R

Other Requirements: Nextflow, Slurm Workload Manager, Environment Modules, Apptainer, Conda

License: Creative Commons 4.0

DOI: https://doi.org/10.48546/workflowhub.workflow.1160.2

3. GitHub for software:

Project name: demuxSNP

Project homepage: https://github.com/michaelplynch/demuxSNP/tree/main [45]

Operating system(s): Windows, MacOS, Linux

Programming language: R

Other requirements: N/A

License: GNU GPL 3

PID:swh:1:snp:277a881b370531cd76c6e2235be60e3fb23f2b87

4. GitHub for benchmarking datasets:

Project name: demuxSNP-benchmarking-datasets

Project homepage: https://github.com/michaelplynch/demuxSNP-benchmarking-datasets [46]

Operating system(s): Windows, MacOS, Linux

Programming language: R

Other requirements: N/A

License: GNU GPL 3

PID: swh:1:snp:430acb307f302cec54e28fd1f3a086930b14f517

5. GitHub for figures:

Project name: demuxSNP-paper-figures

Project homepage: https://github.com/michaelplynch/demuxSNP-paper-figures [47]

Operating system(s): Windows, MacOS, Linux

Programming language: R

Other requirements: N/A

License: GNU GPL 3

PID: swh:1:snp:b0395ee3802e07f0a3b5142f9bb1fcffa42c64de

## Additional Files

**Supplementary Fig. S1**. Alluvial plot comparing ground truth with souporcell and demuxSNP assignment at 45% doublets.

**Supplementary Fig. S2**. HTOreader cluster assignment.

**Supplementary Fig. S3**. Doublet assignment considerations.

**Supplementary Fig. S4**. Distance heatmap for HTODemux Negative and Hashtag2 groups.

**Supplementary Fig. S5**. Accuracy for demuxSNP and souporcell depending on number of genes used to subset SNP list.

**Supplementary Table S1**. Demultiplexing summary statistics for a sample of 14 datasets.

## Abbreviations

scRNA-seq: single-cell RNA sequencing; SNP: single-nucleotide polymorphism; ARI: adjusted Rand index; QC: quality control; UMI: unique molecular identifier.

## Competing Interests

The authors have declared no competing interests.

## Supplementary Material

giae090_Supplementary_Files

giae090_Authors_Response_To_Reviewer_Comments

giae090_GIGA-D-24-00194_Original_Submission

giae090_GIGA-D-24-00194_Revision_1

giae090_GIGA-D-24-00194_Revision_2

giae090_Response_to_Reviewer_Comments_Original_Submission

giae090_Reviewer_1_Report_Original_Submission Lei Li -- 6/19/2024

giae090_Reviewer_1_Report_Revision_1 Lei Li -- 9/30/2024

giae090_Reviewer_2_Report_Original_Submission Haynes Heaton, MD -- 6/30/2024

giae090_Reviewer_2_Report_Revision_1 Haynes Heaton, MD -- 10/10/2024

## Data Availability

Raw and processed multiplexed sequencing data generated in this study (renal cell cancer dataset) are available from the Gene Expression Omnibus (GEO) accession GSE267835. Processed benchmarking data for 5–50% doublets are accessible as SingleCellExperiment objects through an R data package [46].
