# Supplementary material for: demuxSNP: supervised demultiplexing single-cell RNA sequencing using cell hashing and SNPs
Source: Gigascience. 2024 Nov 28;13:giae090. doi: 10.1093/gigascience/giae090 (PMC11604057; doi:10.1093/gigascience/giae090)
Supplement: giae090_Supplementary_Files [file giae090_supplementary_files.zip › supplementary figures.pdf]

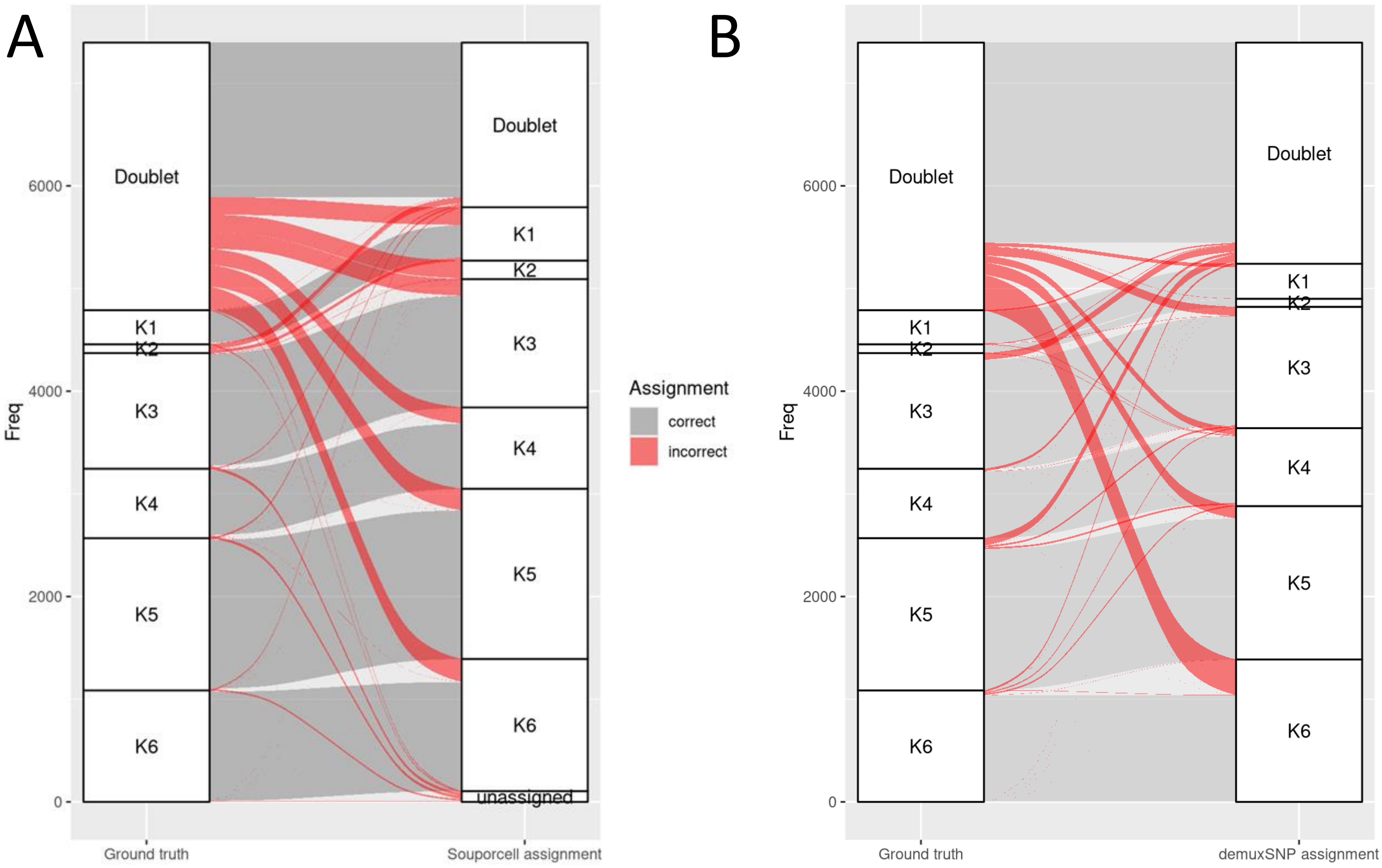

Supplementary Figure 1. (A) Comparison of souporcell assignment with ground truth. K2 group is mostly misassigned as Doublet. (B) Comparison of demuxSNP assignment with ground truth.
Alt text: Assignments for souporcell (A) and demuxSNP (B) compared with ground truth using alluvial plots.

A

Souporcell labels

HTOreader (hashing) labels	2	3	4	5	1	0	Doublet	unassigned
Hashtag1	1235	2	12	4	3	2	38	143
Hashtag2	2	1	1	163	4	1	109	47
Hashtag3	4	17	2904	5	7	3	50	245
Hashtag4	3	2	10	1636	9	7	55	290
Hashtag5	2	2	4	3	3272	1	35	130
Hashtag6	2	6	1	5	2	3367	47	95
Doublet	168	797	456	249	682	810	2796	165
Negative	58	21	121	87	152	49	30	71

B

HTOreader (multi) labels

HTOreader (hashing) labels	doublet	Hashtag1	Hashtag2	Hashtag3	Hashtag4	Hashtag5	Hashtag6	unassigned
Doublet	2796	168	249	456	682	797	810	165
Hashtag1	0	1235	0	0	0	0	0	204
Hashtag2	0	0	163	0	0	0	0	165
Hashtag3	0	0	0	2904	0	0	0	331
Hashtag4	0	0	0	0	9	0	0	2003
Hashtag5	0	0	0	0	0	2	0	3447
Hashtag6	0	0	0	0	0	0	3367	158
Negative	0	58	87	121	152	21	49	101

Supplementary Figure 2. Hybrid methods may inherit misclassifications from genotype methods. Data shown is from ccRCC application dataset. (A) Cross tabulation of HTOreader (hashing) and souporcell. (B) Cross tabulation of HTOreader (multi) and souporcell assignments.

Alt text: Contingency tables. (A) Comparison of HTOreader hashing with souporcell labels. Due to minority cluster misassignment, a 1:1 match does not exist between the two sets of labels. (B) Comparison of HTOreader hashing with HTOreader multi. Lack of 1:1 matching results in two clusters being misassigned.

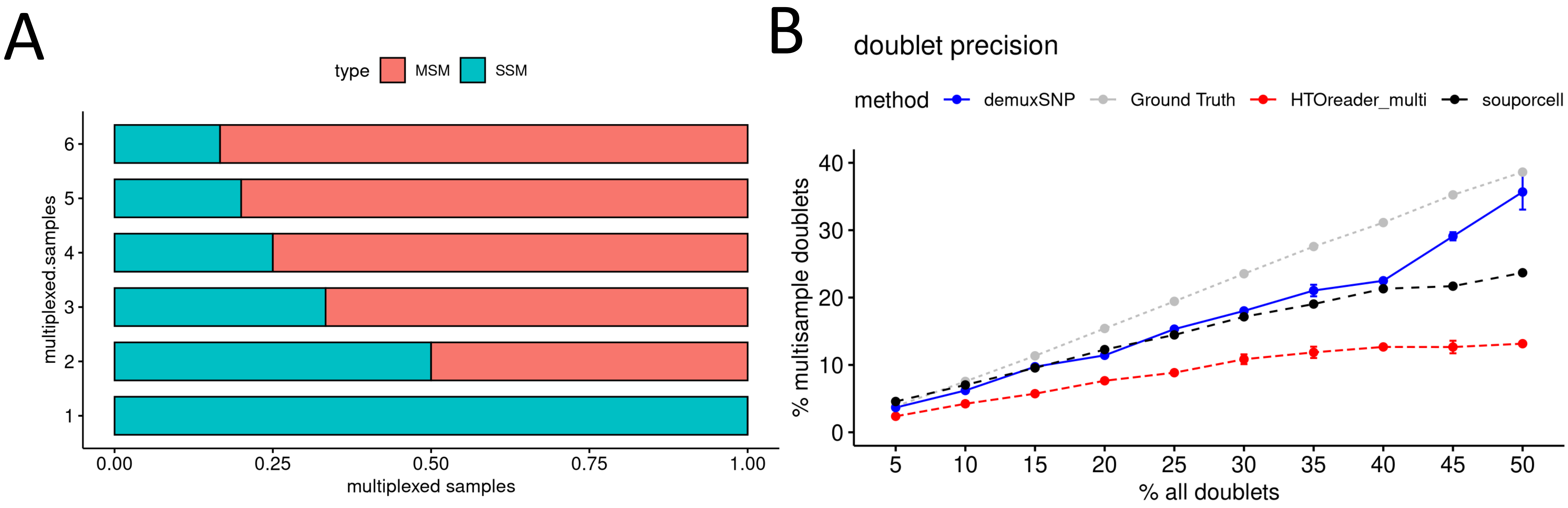

Supplementary Figure 3. Doublet classification. (A) Percentage of multi sample (MSM) vs single sample (SSM) multiplets is dependent on number of multiplexed samples. (B) Methods typically under call multi-sample multiplets relative to ground truth.
Alt text: (A) Bar plot showing approximate percent of single-sample multiplets for one to six multiplexed samples (approximately 1/number of samples). (B) Line graph comparing detected doublets vs true number of multi-sample multiplets for 0-50% total doublets. All methods under call doublets.

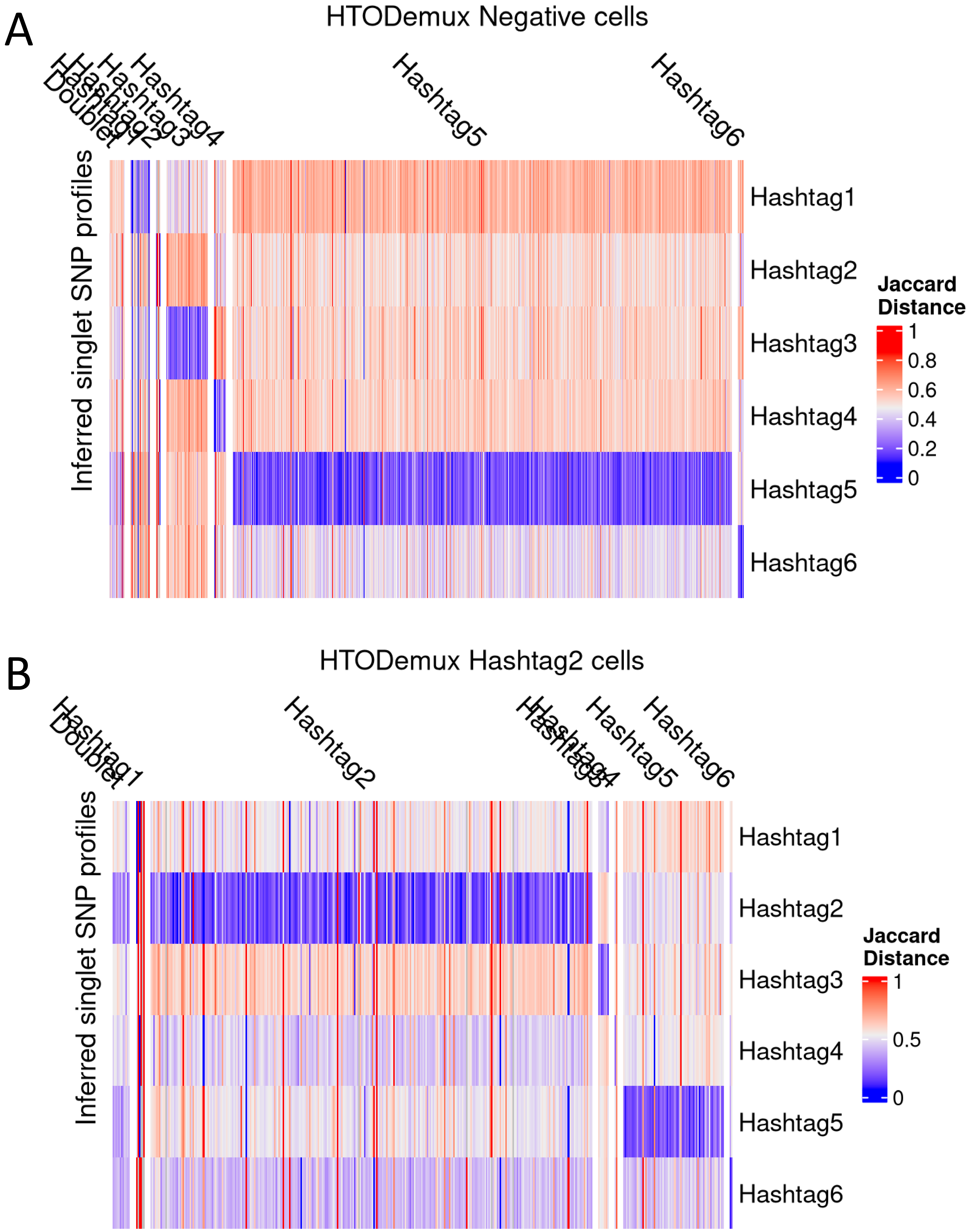

Supplementary Figure 4. Cell reassignment using Jaccard distances between SNPs. (A) Distance between cells in HTODemux Negative group and inferred SNP profiles split by demuxSNP assignment. (B) Distance between cells in HTODemux Hashtag2 group and inferred SNP profiles split by demuxSNP assignment

Alt text: Heatmaps used to show distance between cell's SNPs and inferred SNP profiles for HTODemux Negative and HTODemux Hashtag 2 groups. Cells are reassigned to the group with lowest distance.

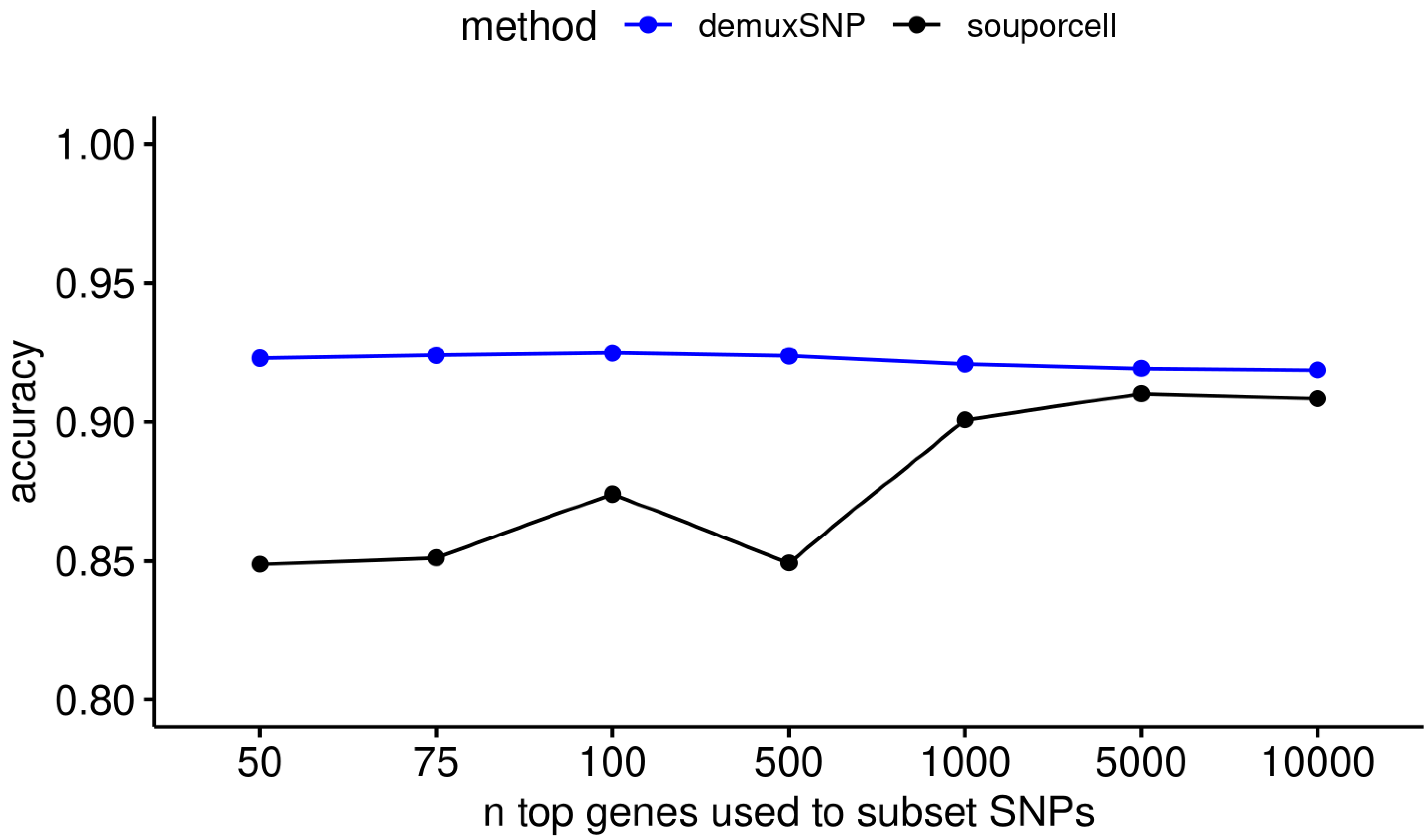

Supplementary Figure 5. Demultiplexing accuracy for demuxSNP and souporcell vs number of genes used to subset SNPs list.
Alt text: line plot with demuxSNP values in blue and souporcell in black. demuxSNP performance remains approximately constant. souporcell performance increases with number of genes/SNPs.
Alt text: Line graph showing accuracy for demuxSNP and souporcell varying the number of genes used to subset SNPs. demuxSNP performance remains stable at 0.92-0.93. souporcell performance increases from approximately 0.85 to 0.91
