# Supplementary material for: demuxSNP: supervised demultiplexing single-cell RNA sequencing using cell hashing and SNPs
Source: Gigascience. 2024 Nov 28;13:giae090. doi: 10.1093/gigascience/giae090 (PMC11604057; doi:10.1093/gigascience/giae090)
Supplement: giae090_Response_to_Reviewer_Comments_Original_Submission [file giae090_response_to_reviewer_comments_original_submission.pdf]

Author's Response To Reviewer Comments

We thank the reviewers for their thoughtful and insightful comments. In response, we updated aspects of the method, revised figures and included additional benchmarking and analysis (Figure 3, Supplementary Figure 5) among other revisions. The manuscript is substantially improved and stronger following the reviewers' suggestions. We have also registered the software with SciCrunch and biotools, and the workflow with workflowhub.eu.

We enclose a point by point response below

Reviewer 1

We thank Reviewer 1 for their time and detailed response. We were pleased to hear that the reviewer found our focus on multi-modal demultiplexing for cell hashing data interesting and that they agree our manuscript demonstrated the method's performance. We have now included additional hybrid benchmarking. Responses to major and minor points are below;

1. I am not surprised that a multi-modal demultiplexing beats single-modal methods across both real and simulated datasets. To my knowledge, there are at least two groups proposed multi-modal demultiplexing approach for cell hashing data. Both were uploaded to bioRxiv last year and get published recently. One called hadge (https://link.springer.com/article/10.1186/s13059-024-03249-z), and another called HTOreader hybrid (https://academic.oup.com/bib/article/25/4/bbae254/7686601), which is discussed by this study. Hadge is a comprehensive framework that integrated popular cell hashing-based and SNP-based methods, allowing for a joint deconvolution by combining best method from each modality. HTOreader hybrid proposed an improved demultiplexing method for cell hashing signals, and then also integrates demultiplexing results from both modality for a better deconvolution in a hybrid fashion. Indeed, this work has implemented different method for the same purpose. I tried both methods, and there're some major updates between bioRxiv version and published version. Thus, even one of them has been discussed, I think it's still necessary to include these two published methods into comparison, to reveal pros and cons of different methods, therefore provide useful information for users to select the method according to their specific experiment configurations.

We thank the reviewer for drawing our attention to the updated version of HTOreader and the hadge pipeline.

We included HTOreader in our SNP benchmarking and discussion. We previously observed that genotype methods (such as souporcell) can misassign a minority cluster when data have class imbalance and high doublet rate. This creates challenges for hybrid methods such as HTOreader which attempt to match hashing and genotype clusters where a 1:1 match does not exist and we also demonstrated this on a real dataset (Supplementary Figure 2). Errors such as mislabelling minority clusters in genotype-free (e.g. souporcell) methods can propagate due to cluster mismatches resulting in significant overall loss of performance (Figure 3A). An advantage of methods such as HTOreader over demuxSNP is their ability to rescue a sample with one completely failed hashtag which we highlighted in the discussion.

We attempted to include hadge, but were unable to run the pipeline. We reached out to the authors, opening an issue on their GitHub (July 4th). Another user responded to our GitHub issue reporting similar challenges. The authors of hadge have not yet responded to the GitHub issue (https://github.com/theislab/hadge/issues/56) which has been open for over two months. Therefore, we were unfortunately unable to include hadge in our benchmarking. Although we were unable to run the hadge pipeline, we expect it may behave similarly to other hybrid methods which leverage existing genotype-free SNP-based methods to rescue experiments with poor hashing calls. Since hadge is a consensus approach, it likely incurs additional computational cost due to running multiple genotype methods.

In the case of demuxSNP, we trained a supervised classifier using SNPs, in contrast to hybrid methods which incorporate cluster labels learned from existing genotype-free SNP methods. We demonstrated that demuxSNP outperforms existing genotype-free or hybrid methods particularly in scenarios with class imbalance and high doublet rate where minority clusters can be misassigned.

2. demuxSNP method picked top N commonly expressed genes for SNP calculation. In the tutorial on Github, the N was set to 100. I am wondering in a more heterozygous dataset, the N = 100 still sufficient or not. Is there a way for users to determine the N for their specific dataset more systematically? Or the authors can show some data to demonstrate that N = 100 is robust across different datasets?

We agree and thank the reviewer for this suggestion. The original model relied heavily on subsetting SNPs to an arbitrary value to minimise missing data. We updated the model and included an additional analysis (Supplementary Figure 5) to demonstrate that the model is stable across a range of N.

Supplementary Figure 5 compared the overall classification accuracy of demuxSNP and souporcell when SNPs are subset to N top genes from 50 to 10,000. The performance of demuxSNP is stable across this range, and only drops slightly (~1%) even when the number of genes used to subset is increased to 10,000.

The increase in stability was achieved by two modifications.
We updated the Jaccard distance calculation so missing data is not included. This was a previous limitation which needed to be addressed given that sparse and missing data are inherent in SNP calls in scRNAseq. Secondly, instead of classifying based on k-nearest neighbours, the SNP profile of each cluster was inferred using the multivariate mode allowing a less sparse representation of the genotype of each singlet and doublet group and labels are assigned to their nearest-neighbour. This allowed for overall improved performance of the model as well as reduced dependence on model parameters including N, the number of genes used to subset SNPs.

3. The dataset GSE267835 is private. Please provide reviewer token in the Data Availability statement during submission process.

We sincerely apologise for this inconvenience. The processed data can be accessed using reviewer token ‘sfczccmevxmtvoh’.

According to the GEO rules for private repositories, SRA sequence records cannot currently be accessed by reviewer token, however, we are happy to arrange transfer of the data if the reviewer wishes to rerun the pipeline. The GEO and SRA record will become public on publication of the manuscript.

4. Color of uncertain cells in Fig1-B is a bit misleading cause in Fig1-A the same color was used to represent "background staining". Even A and B and different panels, however, a big black arrow makes readers thought they're the same data. Therefore, change the color of uncertain cells into another color would be good to avoid confusions.

We thank the reviewer for pointing this out. We have revised the colour scheme and layout of the figure to better convey the workflow.

5. In Fig2-A and B, what are the units for the X axis? Are they log2 or log2 hashtag counts? Please add that information to the figure and legend.

The x-axis units are the natural log of the hashing counts. The figure and legend were updated to specify

this..

6. For Fig-2 C and D, please use the formal spell of names of existing methods like you did in Fig2E.

We have updated the figures to reflect the formal spelling of existing methods..

7. Please add line numbers to the draft for reviewers' convenience

We apologise for the inconvenience and have added line numbers to the revised manuscript.

8. Some minor format issues exist. For example, the "Result" section should a header format instead of normal text.

We thank the reviewer for pointing this out. This has now been fixed along with other minor formatting issues.

Reviewer 2

We are grateful to Reviewer 2 for their time and thoughtful review. We provide a point by point response below.

For figure 2 this is mostly this is good for recovering low hash quality cells. Although because the low quality hashing has been simulated in order to have a ground truth to compare to, it is unclear if this simulation method or amount realistic? Does it compare to % unassigned from real datasets?

Data were simulated using established, previously published approaches. The SNP simulation leveraged methods by Weber et al (DOI: https://doi.org/10.1093/gigascience/giab062). For hashing simulation, the strategy used (attenuating the true signal) was described by Klein (DOI: https://doi.org/10.1093%2Fbioinformatics%2Fbtad481) when they benchmarked demuxmix. Our study combined these two strategies, to maintain more subtle patterns in the data such as correlations between SNP quality and hashing quality that may be challenging to capture using purely simulated data e.g. drawing hashing data from a statistical distribution.

We believe that the simulated data are realistic and the % unassigned compare well to real datasets.
The % negatives for HTODemux in our benchmarks are 3.6% and 7.9% for the high and low quality hashing scenarios respectively. To confirm these are realistic, we partnered with Harvard Chan Bioinformatics Core (https://bioinformatics.sph.harvard.edu/) and asked them to provide summary statistics from randomly selected multiplexed datasets generated by their core facility. They provide demultiplexing statistics on 14 multiplexed scRNAseq studies which are included in the manuscript (Supplementary Table 1). Demultiplexing statistics provided are from data after low RNA quality cells were removed, and so should be interpreted as a reliable indicator of the number of negative/unassigned cells with high RNA quality. Twelve datasets were demultiplexed using hashing based method HTODemux, and had % negatives ranging from 1-17%, with mean 5%, median 5%.
The real dataset used in this study (renal cell cancer) had 10.3% negatives with HTODemux demultiplexing. The simulated data for HTODemux falls within this range.
The % negatives are method dependent (Figure 2D). The highest % negatives in our low quality hashing benchmarking was approximately 30% for BFF_raw. In other published benchmarking studies, % negatives have been reported as high as approximately 50% (estimate from BFF_raw, Figure 2 from Howitt et al.,

DOI: https://doi.org/10.1093/nargab/lqad086).

For figure 3 my main issue is why would souporcell out perform demuxSNP at any % doublets? Souporcell is using strictly less information than demuxSNP because it does not assume hashtags. Ideally this would be fixed or at the very lease an adequate explanation given.

We agree with the reviewer. Thank you. This was an insightful observation and we are grateful that the reviewer asked us to investigate this as it improved our study.

Using more data should result in improved performance. Although demuxSNP uses two modalities instead of one modality used by souporcell, souporcell outperformed demuxSNP because the Jaccard distance metric computations did not account for missing data and the error introduced was greater with more SNPs.

To address this, we updated the Jaccard distance calculation to exclude missing data in the distance calculations, thus improving handling of sparse/missing data. Additionally, instead of using k-nearest neighbours, cells are classified based on nearest multivariate mode for singlet and doublet clusters, also maximising the data available for comparing SNP similarity. We updated the methods section with these details. This has resulted in consistent improved overall performance (Figure 3A) compared to souporcell and contributed to the overall robustness of the method.

Comments on methods:
"SNPs are filtered to those located within genes expressed across most cells in the
dataset" and "SNPs with few reads across cells in the dataset are removed." -- Can I get numbers on this? If you require say 50% of cells to express a SNP locus, it will throw out a huge amount of the still informative SNPs. I find that utilizing as much of the data as possible is generally better. I assume this is done because of the KNN method which will require high overlap in SNPs between cells being compared.

We agree this was confusing. This was a limitation in our approach and has been addressed in response to reviewers’ comments. Previously, the threshold was 80%, resulting in less missing data but significantly fewer SNPs being used for classification. This is no longer a necessity based on the model updates we described above.

We carried out additional testing to quantify the impact of the number of genes used to subset the SNPs list (Supplementary Figure 5). We observed that demuxSNP’s performance remained stable across the range of 50-10,000 genes, although there is minimal loss when genes exceed 500. Souporcell does benefit from more data (Supplementary Figure 5).

Previous evidence from the literature also suggests that using fewer SNPs may yield satisfactory performance, e.g. Kang et al. (DOI: https://doi.org/10.1038/nbt.4042) demonstrated that Demuxlet could identify singlets and doublets with 97% and 92% accuracy respectively from 50 SNPs in simulated testing, Weber et al. (DOI: https://doi.org/10.1093/gigascience/giab062) tested different SNP filtering strategies which showed minor loss of performance.

"Labels from high confidence singlets along with simulated doublets used to train KNN classifier and predict negative/uncertain cells." Why a KNN model here. Genotype data is not euclidean. Each SNP locus for each cell should be drawn as a binomial with underlying p=0+some error (homozygous ref) p=0.5+/- some error (heterozygous), or p=1.0-some error
A statistical model would be more appropriate for this.

We agree that using the alternative fraction format of the SNP data would not be considered Euclidean, and

several existing models do indeed successfully use a statistical model in this way (e.g. souporcell and Vireo DOI: https://doi.org/10.1186/s13059-019-1865-2). Instead, we leverage the consensus format from varTrix which we then recode to a binary form, with the limitation that the heterozygous alt case is not differentiated from the homozygous alt which the reviewer correctly identified and we discuss below.

"To leverage classification techniques applicable to binary data, SNP status is recoded to absent/present (1,0) and k-nearest-neighbour classification (KNN) [31] is performed using Jaccard coefficient." Ah, so you force the data to be euclidean, but this does not take full advantage of the data. One problem with this will be when two individuals are related. For SNP loci of a parent/child there are many cases where this potentially could have disambiguated them but won't because one individual is heterozygous (so snp present) and the other is homozygous alt (still snp present).

We agree with the reviewer, the heterozygous case is not differentiated from the homozygous case here. The model has been developed based on classification on data with two states (alt SNP absent or present). While we were able to consider missing data in the distance calculation, we have not been able to accommodate consideration of heterozygous alt vs. homozygous alt SNP loci in this version of the model. We have updated the documentation and software messaging to make it clear to users that the method is not suited to such applications where multiplexed samples are closely related. These changes will be reflected in the package Github and in the next Bioconductor package release v3.20 on October 30th.

General comments: these are small nitpicks
The primary failure modes of genotype demultiplexing in no particular order are 1. small number of cells in a minority cluster 2. large number of individuals multiplexed together. and 3. large number of doublets causing lots of noise in the statistical models. The authors have adequately addressed improvements in 1 and 3. However, I think the paper would be stronger if it also did experiments with >30 individuals multiplexed together. For 3, I think further discussion is merited on the tradeoffs of hyperloading scRNAseq protocols including the # of quality singletons vs loading rate and multiplet rate and how many multiplets escape detection. Experiment designers want to maximize the number of singletons while minimizing the number of doublets that escape detection and harm downstream analyses. 10x genomics gives the ballpark doublet % to be expected as 1% per 1000 cells recovered. But this is a poisson loading process, so the true effect is not linear. The authors test up to 50% doublets (which is good to test), and some experimenters do attempt to load enough to recover 50k cells from a single lane, but I doubt that would be a recommended loading level for downstream analysis unless the doublet detection is highly effective.

We thank the reviewer for this insightful analysis on the challenges of genetic demultiplexing. Relating to failure mode 2 (large numbers of individuals), demuxSNP relies on hashing to determine the genotypes associated with each sample, and so will always be limited by the number of samples which can be multiplexed using hashing methods. For example, 10X Genomics' website describes an upper limit of 12 multiplexed samples per experiment (Single Cell Gene Expression with Cell Multiplexing technology - Official 10x Genomics Support) so this provides an upper limit for where demuxSNP can be used. We highlight in our discussion that this is a limitation of our method and an application for methods such as souporcell which are not dependent on hashing.

Relating to failure mode 3 (% doublets), we agree that the ability to detect doublets is key in optimising experimental design. To this end, we added to the results and discussion outlining various considerations for experimental design and algorithm performance. These include proportions of single-sample vs multi-sample multiplets depending on number of multiplexed samples (Supplementary Figure 3) and precision and recall for doublet detection for benchmarked methods (Figure 3C). We also discussed the trade off between high doublet precision and recall in terms of downstream analysis, from which we concluded that doublet recall is sufficiently low to warrant continued use of downstream doublet detection methods, even in experiments with few expected single-sample multiplets.
We also agree that 50% doublet rate is likely rarely targeted in experiments, however we included it to observe behaviours in our benchmarking which are dependent on doublet rate but are not dependent on a

specific doublet threshold. We highlighted this in our discussion also. For example, in our simulated benchmarking, the minority cluster was misassigned at >40% doublets, whereas for the renal cell cancer application dataset, the minority cluster was missed at 16-24% doublets (estimated based on expected doublet rate from recovered cells).
