# Supplementary material for: demuxSNP: supervised demultiplexing single-cell RNA sequencing using cell hashing and SNPs
Source: Gigascience. 2024 Nov 28;13:giae090. doi: 10.1093/gigascience/giae090 (PMC11604057; doi:10.1093/gigascience/giae090)
Supplement: giae090_Reviewer_1_Report_Original_Submission — Lei Li -- 6/19/2024 [file giae090_reviewer_1_report_original_submission.pdf]

Reviewer Report

Title: demuxSNP: supervised demultiplexing single-cell RNA sequencing using cell hashing and SNPs

Version: Original Submission **Date:** 6/19/2024

Reviewer name: Lei Li

Reviewer Comments to Author:

Lynch et. al developed demuxSNP, a supervised demultiplexing approach for single-cell cell hashing data in a multi-modal (hashtag expression and SNP profiles) fashion. They utilized a probabilistic method to infer sample identities of cells using cell hashing modality, and then build a KNN model using SNPs of high cofinance ones from previous step. They then use this KNN model to predict cell identities for cells assigned as uncertain or negative by cell hashing.They have demonstrated the performance through a comparison with existing single-modal methods using both real data and simulated data. They have published an R package for the research community. It is interesting and encouraging to see another study focuses on multi-modal demultiplexing for cell hashing data. Below are some major and minor points from my side:1. I am not surprised that a multi-modal demultiplexing beats single-modal methods across both real and simulated datasets. To my knowledge, there are at least two groups proposed multi-modal demultiplexing approach for cell hashing data. Both were uploaded to bioRxiv last year and get published recently. One called hadge (https://link.springer.com/article/10.1186/s13059-024-03249-z), and another called HTOreader hybrid (https://academic.oup.com/bib/article/25/4/bbae254/7686601), which is discussed by this study. Hadge is a comprehensive framework that integrated popular cell hashing-based and SNP-based methods, allowing for a joint deconvolution by combining best method from each modality. HTOreader hybrid proposed an improved demultiplexing method for cell hashing signals, and then also integrates demultiplexing results from both modality for a better deconvolution in a hybrid fashion. Indeed, this work has implemented different method for the same purpose. I tried both methods, and there're some major updates between bioRxiv version and published version. Thus, even one of them has been discussed, I think it's still necessary to include these two published methods into comparison, to reveal pros and cons of different methods, therefore provide useful information for users to select the method according to their specific experiment configurations.2. demuxSNP method picked top N commonly expressed genes for SNP calculation. In the tutorial on Github, the N was set to 100. I am wondering in a more heterozygous dataset, the N = 100 still sufficient or not. Is there a way for users to determine the N for their specific dataset more systematically? Or the authors can show some data to demonstrate that N = 100 is robust across different datasets?3. The dataset GSE267835 is private. Please provide reviewer token in the Data Availability statement during submission process.4. Color of uncertain cells in Fig1-B is a bit misleading cause in Fig1-A the same color was used to represent "background staining". Even A and B and different panels, however, a big black arrow makes readers thought they're the same data. Therefore, change the color of uncertain cells into another color would be good to avoid confusions.5. In Fig2-A and B, what are the units for the X axis? Are they log2 or log2 hashtag counts? Please add that information to the figure and legend.6. For Fig-2 C and D, please use the formal spell of

names of existing methods like you did in Fig2E.7. Please add line numbers to the draft for reviewers' convenience8. Some minor format issues exist. For example, the "Result" section should a header format instead of normal text.

Level of Interest

Please indicate how interesting you found the manuscript: Choose an item.

Quality of Written English

Please indicate the quality of language in the manuscript: Choose an item.

Declaration of Competing Interests

Please complete a declaration of competing interests, considering the following questions:

- Have you in the past five years received reimbursements, fees, funding, or salary from an organisation that may in any way gain or lose financially from the publication of this manuscript, either now or in the future?
- Do you hold any stocks or shares in an organisation that may in any way gain or lose financially from the publication of this manuscript, either now or in the future?
- Do you hold or are you currently applying for any patents relating to the content of the manuscript?
- Have you received reimbursements, fees, funding, or salary from an organization that holds or has applied for patents relating to the content of the manuscript?
- Do you have any other financial competing interests?
- Do you have any non-financial competing interests in relation to this paper?

If you can answer no to all of the above, write 'I declare that I have no competing interests' below. If your reply is yes to any, please give details below.

I declare that I have no competing interests

I agree to the open peer review policy of the journal. I understand that my name will be included on my report to the authors and, if the manuscript is accepted for publication, my named report including any attachments I upload will be posted on the website along with the authors' responses. I agree for my report to be made available under an Open Access Creative Commons CC-BY license (http://creativecommons.org/licenses/by/4.0/). I understand that any comments which I do not wish to be included in my named report can be included as confidential comments to the editors, which will not be published.

Choose an item.

To further support our reviewers, we have joined with Publons, where you can gain additional credit to further highlight your hard work (see: https://publons.com/journal/530/gigascience). On publication of this paper, your review will be automatically added to Publons, you can then choose whether or not to claim your Publons credit. I understand this statement.

Yes Choose an item.
