# Supplementary material for: demuxSNP: supervised demultiplexing single-cell RNA sequencing using cell hashing and SNPs
Source: Gigascience. 2024 Nov 28;13:giae090. doi: 10.1093/gigascience/giae090 (PMC11604057; doi:10.1093/gigascience/giae090)
Supplement: giae090_Reviewer_2_Report_Original_Submission — Haynes Heaton, MD -- 6/30/2024 [file giae090_reviewer_2_report_original_submission.pdf]

Reviewer Report

Title: demuxSNP: supervised demultiplexing single-cell RNA sequencing using cell hashing and SNPs

Version: Original Submission **Date:** 6/30/2024

Reviewer name: Haynes Heaton, MD

Reviewer Comments to Author:

demuxSNP is a tool for combining the demultiplexing capabilities of hashtagging and SNP based genotype demultiplexing of scRNAseq with cells from individuals mixed for cost savings and batch effect reduction. The authors test this method in comparison with other methods for either hashtag demultiplexing or genotype based demultiplexing individually and show improvements in recovering cells not confidently assigned via hashtagging as well as overcoming cases where genotype demultiplexing fails.comments on results:For figure 2 this is mostly this is good for recovering low hash quality cells. Although because the low quality hashing has been simulated in order to have a ground truth to compare to, it is unclear if this simulation method or amount realistic? Does it compare to % unassigned from real datasets?For figure 3 my main issue is why would souporcell out perform demuxSNP at any % doublets? Souporcell is using strictly less information than demuxSNP because it does not assume hashtags. Ideally this would be fixed or at the very lease an adequate explanation given.Comments on methods:"SNPs are filtered to those located within genes expressed across most cells in thedataset" and "SNPs with few reads across cells in the dataset are removed." -- Can I get numbers on this? If you require say 50% of cells to express a SNP locus, it will throw out a huge amount of the still informative SNPs. I find that utilizing as much of the data as possible is generally better. I assume this is done because of the KNN method which will require high overlap in SNPs between cells being compared."Labels from high confidence singlets along with simulated doublets used to train KNNclassifier and predict negative/uncertain cells." Why a KNN model here. Genotype data is not euclidean. Each SNP locus for each cell should be drawn as a binomial with underlying p=0+some error (homozygous ref) p=0.5+/- some error (heterozygous), or p=1.0-some errorA statistical model would be more appropriate for this."To leverage classification techniques applicable to binary data, SNP status is recoded toabsent/present (1,0) and k-nearest-neighbour classification (KNN) [31] is performed usingJaccard coefficient." Ah, so you force the data to be euclidean, but this does not take full advantage of the data. One problem with this will be when two individuals are related. For SNP loci of a parent/child there are many cases where this potentially could have disambiguated them but wont because one individual is heterozygous (so snp present) and the other is homozygous alt (still snp present).General comments: these are small nitpicksThe primary failure modes of genotype demultiplexing in no particular order are 1. small number of cells in a minority cluster 2. large number of individuals multiplexed together. and 3. large number of doublets causing lots of noise in the statistical models. The authors have adequately addressed improvements in 1 and 3. However, I think the paper would be stronger if it also did experiments with >30 individuals multiplexed together. For 3, I think further discussion is merited on the tradeoffs of hyperloading scRNAseq protocols including the # of quality singletons vs loading rate and multiplet rate and how many multiplets escape detection.

Experiment designers want to maximize the number of singletons while minimizing the number of doublets that escape detection and harm downstream analyses. 10x genomics gives the ballpark doublet % to be expected as 1% per 1000 cells recovered. But this is a poisson loading process, so the true effect is not linear. The authors test up to 50% doublets (which is good to test), and some experimenters do attempt to load enough to recover 50k cells from a single lane, but I doubt that would be a recommended loading level for downstream analysis unless the doublet detection is highly effective.

Level of Interest

Please indicate how interesting you found the manuscript: Choose an item.

Quality of Written English

Please indicate the quality of language in the manuscript: Choose an item.

Declaration of Competing Interests

Please complete a declaration of competing interests, considering the following questions:

- Have you in the past five years received reimbursements, fees, funding, or salary from an organisation that may in any way gain or lose financially from the publication of this manuscript, either now or in the future?
- Do you hold any stocks or shares in an organisation that may in any way gain or lose financially from the publication of this manuscript, either now or in the future?
- Do you hold or are you currently applying for any patents relating to the content of the manuscript?
- Have you received reimbursements, fees, funding, or salary from an organization that holds or has applied for patents relating to the content of the manuscript?
- Do you have any other financial competing interests?
- Do you have any non-financial competing interests in relation to this paper?

If you can answer no to all of the above, write 'I declare that I have no competing interests' below. If your reply is yes to any, please give details below.

I declare that I have no competing interests

I agree to the open peer review policy of the journal. I understand that my name will be included on my report to the authors and, if the manuscript is accepted for publication, my named report including any attachments I upload will be posted on the website along with the authors' responses. I agree for my report to be made available under an Open Access Creative Commons CC-BY license (http://creativecommons.org/licenses/by/4.0/). I understand that any comments which I do not wish to

be included in my named report can be included as confidential comments to the editors, which will not be published.

Choose an item.

To further support our reviewers, we have joined with Publons, where you can gain additional credit to further highlight your hard work (see: https://publons.com/journal/530/gigascience). On publication of this paper, your review will be automatically added to Publons, you can then choose whether or not to claim your Publons credit. I understand this statement.

Yes Choose an item.
