# Supplementary material for: demuxSNP: supervised demultiplexing single-cell RNA sequencing using cell hashing and SNPs
Source: Gigascience. 2024 Nov 28;13:giae090. doi: 10.1093/gigascience/giae090 (PMC11604057; doi:10.1093/gigascience/giae090)
Supplement: giae090_Authors_Response_To_Reviewer_Comments [file giae090_authors_response_to_reviewer_comments.pdf]

Author's Response To Reviewer Comments

We thank the editor and curators for their time in reviewing the manuscript. We have added the software references and citations as requested by the data curators.
